# Comet assay for quantification of the increased DNA damage burden in primary human chondrocytes with aging and osteoarthritis

**DOI:** 10.1111/acel.13698

**Published:** 2022-08-22

**Authors:** Michaela E. Copp, Susan Chubinskaya, Daniel N. Bracey, Jacqueline Shine, Garrett Sessions, Richard F. Loeser, Brian O. Diekman

**Affiliations:** ^1^ Joint Department of Biomedical Engineering University of North Carolina at Chapel Hill, Chapel Hill and North Carolina State University Raleigh North Carolina USA; ^2^ Thurston Arthritis Research Center University of North Carolina at Chapel Hill Chapel Hill North Carolina USA; ^3^ Comparative Medicine Institute North Carolina State University Raleigh North Carolina USA; ^4^ Department of Pediatrics Rush University Medical Center Chicago Illinois USA; ^5^ Department of Orthopaedics University of North Carolina Chapel Hill North Carolina USA; ^6^ Department of Cell Biology & Physiology University of North Carolina Chapel Hill North Carolina USA; ^7^ Division of Rheumatology, Allergy, and Immunology University of North Carolina Chapel Hill North Carolina USA

**Keywords:** arthritis, cartilage, single‐cell gel electrophoresis

## Abstract

It is known that chondrocytes from joints with osteoarthritis (OA) exhibit high levels of DNA damage, but the degree to which chondrocytes accumulate DNA damage during “normal aging” has not been established. The goal of this study was to quantify the DNA damage present in chondrocytes obtained from cadaveric donors of a wide age range, and to compare the extent of this damage to OA chondrocytes. The alkaline comet assay was used to measure the DNA damage in normal cartilage from the ankle (talus) and the knee (femur) of cadaveric donors, as well as in OA chondrocytes obtained at the time of total knee replacement. Chondrocytes from younger donors (<45 years) had less DNA damage than older donors (>70 years) as assessed by the percentage of DNA in the comet “tail”. In donors between 50 and 60 years old, there was increased DNA damage in chondrocytes from OA cartilage as compared to cadaveric. Talar chondrocytes from 23 donors between the ages of 34 and 78 revealed a linear increase in DNA damage with age (*R* ^2^ = 0.865, *p* < 0.0001). A “two‐tailed” comet assay was used to demonstrate that most of the accumulated damage is in the form of strand breaks as opposed to alkali‐labile base damage. Chondrocytes from young donors required 10 Gy irradiation to recapitulate the DNA damage present in chondrocytes from older donors. Given the potential for DNA damage to contribute to chondrocyte dysfunction and senescence, this study supports the investigation of mechanisms by which hypo‐replicative cell types accumulate high levels of damage.

## Graphical abstract

Chondrocytes accumulate a substantial burden of DNA damage during aging. As measured by the alkaline comet assay, there is a linear increase from 4th to 8th decade of life. The damage in tissue from donors >70 years old is similar to cartilage taken at the time of joint replacement for osteoarthritis.

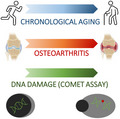

**Abbreviations**
BER: base excision repair
DNA: deoxyribonucleic acid
DSB: double‐strand breaks
IR: Irradiation
MMS: methyl methanesulfonate
OA: osteoarthritis
SSB: single‐strand breaks

## 1 INTRODUCTION

Osteoarthritis (OA) is a degenerative disorder characterized by joint pain and the progressive degradation of articular cartilage and other tissues of the joint. Aging is the strongest risk factor for the development of OA, but the mechanisms that drive this relationship remain unclear (Loeser et al., 2016). Previous studies have shown that OA chondrocytes exhibit high levels of DNA damage and that this may contribute to heterogeneous gene expression and dysfunction (Chen et al., 2008; Rose et al., 2012). However, the degree to which chondrocytes accumulate DNA damage during “normal aging” has not been reported in the literature. Chondrocytes and other hypo‐replicative cells may be particularly susceptible to acquiring sites of persistent DNA damage with age, as they cannot take advantage of the efficient and accurate DNA repair mechanisms that are restricted to S phase (Arnoult et al., 2017; Reid et al., 2021). It is important to quantify this burden, as DNA damage is a central mediator of numerous aspects of aging, including cell senescence (Schumacher et al., 2021; Yousefzadeh et al., 2021). A widely established method for measuring DNA damage in individual cells is the comet assay, which is sufficiently sensitive to serve as an effective biomonitoring tool for assessing the effect of various environmental exposures (Milic et al., 2021). The alkaline comet assay uses gel electrophoresis of single nuclei and fluorescence microscopy to visualize damaged DNA in the “comet tail” as compared to intact DNA in the “comet head” (Olive & Banath, 2006). The comet tail is caused by relaxation of supercoiled DNA loops due to strand breaks, which results in greater migration through the agarose gel when an electric field is applied. The alkaline comet assay detects single‐strand breaks (SSBs) and double‐strand breaks (DSBs), as well as a basic sites and other forms of damage that can be converted into strand breaks under alkaline conditions (Olive & Banath, 2006). To gain additional information on the type of damage present in a given cell, a “two‐tailed” version of the comet uses sequential electrophoresis steps in orthogonal directions under neutral pH and then alkaline pH buffer conditions (Cortes‐Gutierrez et al., 2017). The goal of this study was to use the comet assay to quantify the DNA damage present in chondrocytes obtained from cadaveric donors of a wide age range, and to compare the extent of this damage to OA chondrocytes taken at the time of joint replacement. Lastly, we treated chondrocytes from young donors with irradiation to identify the dose of DNA damage that was required to recapitulate the baseline levels found in older donors.

## 2 RESULTS

### 2.1 DNA damage increases with age in primary human chondrocytes

Cadaveric donors without a history of OA served as the source of normal cartilage from the ankle (talus) and the knee (femur). Tissue was obtained from organ donors within 24 h of death through the Gift of Hope Organ and Tissue Donor Network (Itasca, IL) and shipped overnight to the University of North Carolina at Chapel Hill (UNC). Tissue was graded according to the 0–4 point Collins scale and only cartilage from regions that were macroscopically normal were used (Muehleman et al., 1997). Cartilage was dissected away from the underlying bone and chondrocytes were isolated by enzymatic digestion with Pronase for 1 h and subsequently with Collagenase P overnight (Forsyth et al., 2002), followed by plating at ~1 × 10^5^ cells/cm^2^ for a recovery period of ~2–7 days before cryopreservation (12,648,010, Thermo Fisher). Cryopreserved cells were thawed and plated for ~2–3 days to recover before performing the comet assay in batches that represented all groups being compared.

**FIGURE 1:**
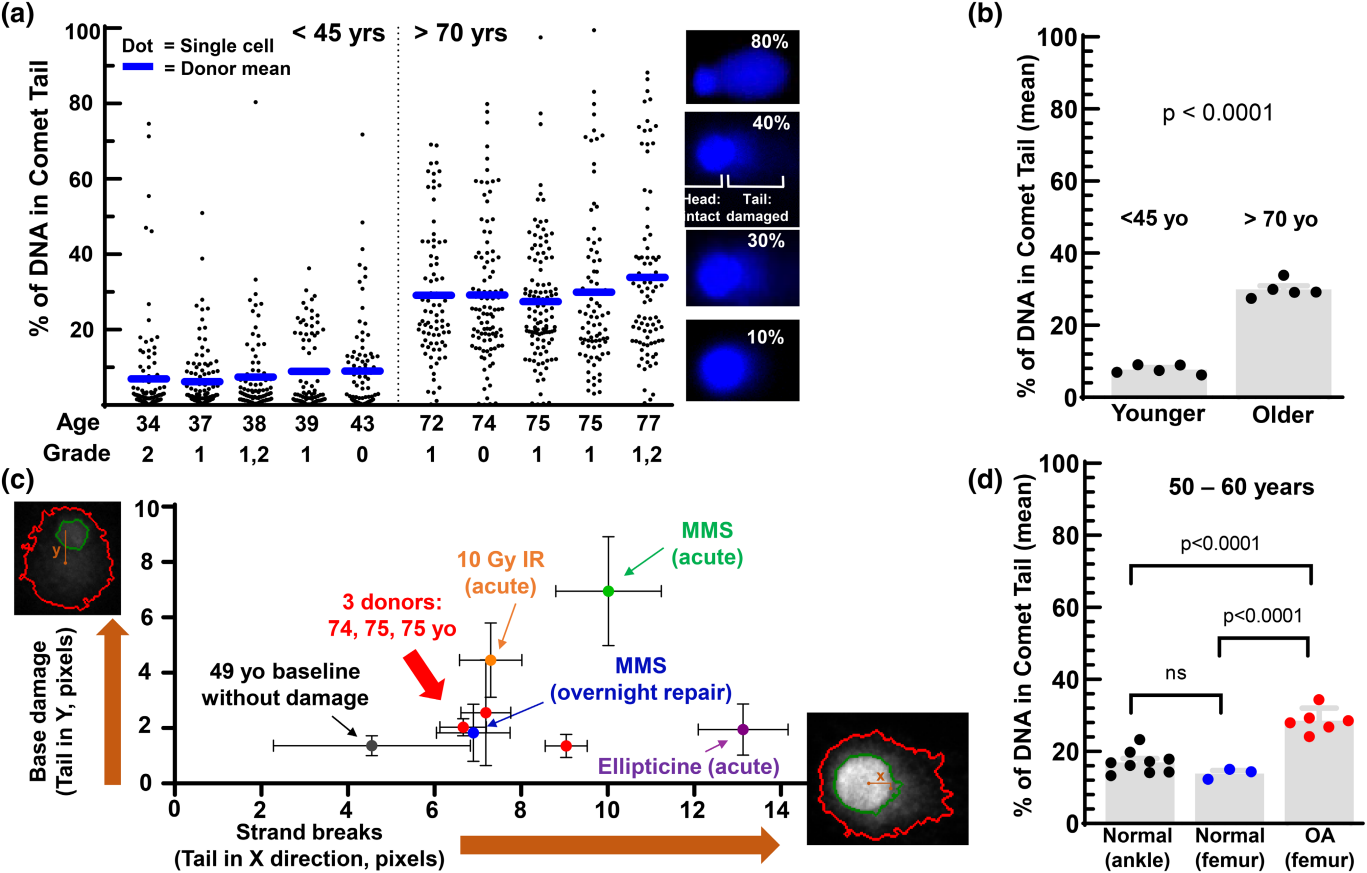
DNA damage in chondrocytes with aging and osteoarthritis. (a) Percent DNA in comet tail for chondrocytes from cadaveric donors of various ages with Collins grade shown. Dots are individual cells, with the mean shown as blue bars. Example cells with given % DNA in tail shown, with wide‐field images in Figure S1. (b) Donor mean % DNA in comet tail for those younger than 45 years of age (mean: 7.7%) and older than 70 years of age (mean: 29.9%). Stats by *t*‐test. (c) Two‐tailed comet using distance between centroid of comet head (green outline) and centroid of entire region including the tail (red outline). Strand breaks show up in x direction under neutral conditions, and base damage is detected under alkaline conditions during the second electrophoresis (y direction). Chondrocytes from 49 yo serve as low damage control and were treated to induce damage. Ellipticine: 1 h with 1 μM; MMS acute: 30 min with 0.5 mM; MMS repair: Treatment removed before overnight repair; IR: 10 Gy irradiation applied to cells in gel on comet slide 15 min before lysis. Mean ± SEM both directions. ~100 cells per group. (d) Cadaveric tissue from donors with no clinical history of OA as compared to cartilage from end‐stage OA at total knee replacement. All donors between 50 and 60 years of age. Mean tail DNA percent in the normal ankle is 17.5%, in normal femur is 13.9%, and in OA tissue is 28.5%. Stats by ANOVA with Tukey's post hoc. Single‐cell analysis for panel D shown in Figure S2.

DNA damage in primary human chondrocytes from young (˂45 years) and older (˃70 years) donors was assessed using the comet assay. Chondrocytes were trypsinized, and 5 × 10^4^ chondrocytes were mixed with 1% Low Melting Agarose (A0701, Millipore Sigma) at a 1:10 volume ratio and applied to a Superfrost slide (12‐550‐15, Thermo Fisher) pre‐coated with 1% normal melting agarose (20–240, Apex). The slides were placed in the lysis solution (2.5 M NaCl, 0.1 M disodium EDTA, 10 mM Tris base, 0.2 M NaOH, 0.1% sodium lauryl sarcosinate, 1% Triton X‐1000, pH 10) overnight at 4°C. After lysis, slides were immersed at 4°C in an alkaline electrophoresis solution (200 mM NaOH, 1 mM disodium EDTA, pH > 13) for 45 min followed by electrophoresis at 1 V/cm and 300 mA for 20 min. Samples were washed twice with dH_2_0, dried and stained with NucBlue™ nuclear stain (R37605; Thermo Fisher Scientific). Approximately 100 randomly selected cells per condition were imaged under an EVOS M5000 microscope (AMF5000; Thermo Fisher Scientific) and analyzed in ImageJ using the OpenComet plugin software. Representative wide‐field images of chondrocyte comets from young, older, and OA donors are provided in Figure S1. Chondrocytes from older donors revealed a wide distribution in the percentage of DNA found in the comet tail, whereas most chondrocytes from younger donors exhibited low or moderate levels of DNA damage (Figure 1a). The driving factor in the distribution of tail DNA percentage was donor age, with a Collins grade between 0 and 2 showing no effect on the tail DNA percentage (Figure 1a). The tail DNA percentage was averaged across all cells for a given donor and grouped according to age, with the older donors showing a significant increase in DNA damage compared to young donors (Figure 1b, unpaired t‐test *p* < 0.0001).

### 2.2 Two‐tailed comet indicates that chondrocytes from older donors harbor damage in the form of strand breaks

We assessed the type of DNA damage in chondrocytes from three >70‐year‐old donors using a two‐tailed comet assay, where strand breaks are represented by a tail in the “x direction” due to a first electrophoresis under neutral pH conditions, and base damage is represented by a tail in the “y direction” due to a second electrophoresis with alkaline pH (the slide is rotated 90° between runs). We followed a published protocol (Cortes‐Gutierrez et al., 2017), with a modification to perform lysis II at 37 degrees to avoid precipitation. A custom script was written using CellProfiler™ to calculate the “x” and “y” distance in pixels between the centroid of the head and the centroid of all stained DNA. The DNA damage present in chondrocytes from older donors is predominantly in the form of strand breaks rather than base damage (Figure 1c). While some investigators suggest that neutral pH conditions detect only DSBs and not SSBs (Cortes‐Gutierrez et al., 2017; Enciso et al., 2009; Lu et al., 2017), here is strong experimental and theoretical support for the interpretation that all strand breaks are detected at neutral pH and alkali conditions additionally detect base damage (Afanasieva & Sivolob, 2018; Collins et al., 2008; Gradzka & Iwanenko, 2005). The data from the irradiation control are consistent with this latter interpretation, as the expected ratio of SSB:DSB is ~20:1 (Roots et al., 1985), and therefore, the abundant SSBs would bias the tails strongly toward the y‐direction if neutral conditions only detected DSBs. As expected given the complexity of damage in response to irradiation (Nikjoo et al., 2001), 10 Gy did cause both strand breaks and base damage. Other controls show that young chondrocytes treated with ellipticine have strand breaks as expected for this DSB‐inducing agent (Pommier et al., 1984), methyl methanesulfonate (MMS) shows acute base damage due to direct alkylation (Wyatt & Pittman, 2006), and overnight recovery after MMS treatment reveals the SSBs that are generated during failed base excision repair (BER) (Wyatt & Pittman, 2006).

### 2.3 Osteoarthritis accelerates DNA damage as compared to age‐matched normal donors

OA cartilage was obtained from intact areas of the femur at the time of total knee replacement surgeries performed at the UNC Medical Center. Tissue was handled in a manner consistent with the cadaveric donors by storing in saline at 4°C for 24–48 h before dissociation and cell isolation. Using donors between 50 and 60 years old, chondrocytes derived from OA cartilage showed higher levels of DNA damage as compared to chondrocytes derived from femoral and ankle cartilage of cadaveric donors (Figure 1d, Tukey's multiple comparison test, *p* < 0.0001; individual cell data in Figure S2). Cadaveric tissue from both the knee and ankle was used to address the possibility that anatomical site may alter the level of DNA damage, but there was no effect (Figure 1d).

### 2.4 Linear increase in DNA damage with age

**FIGURE 2:**
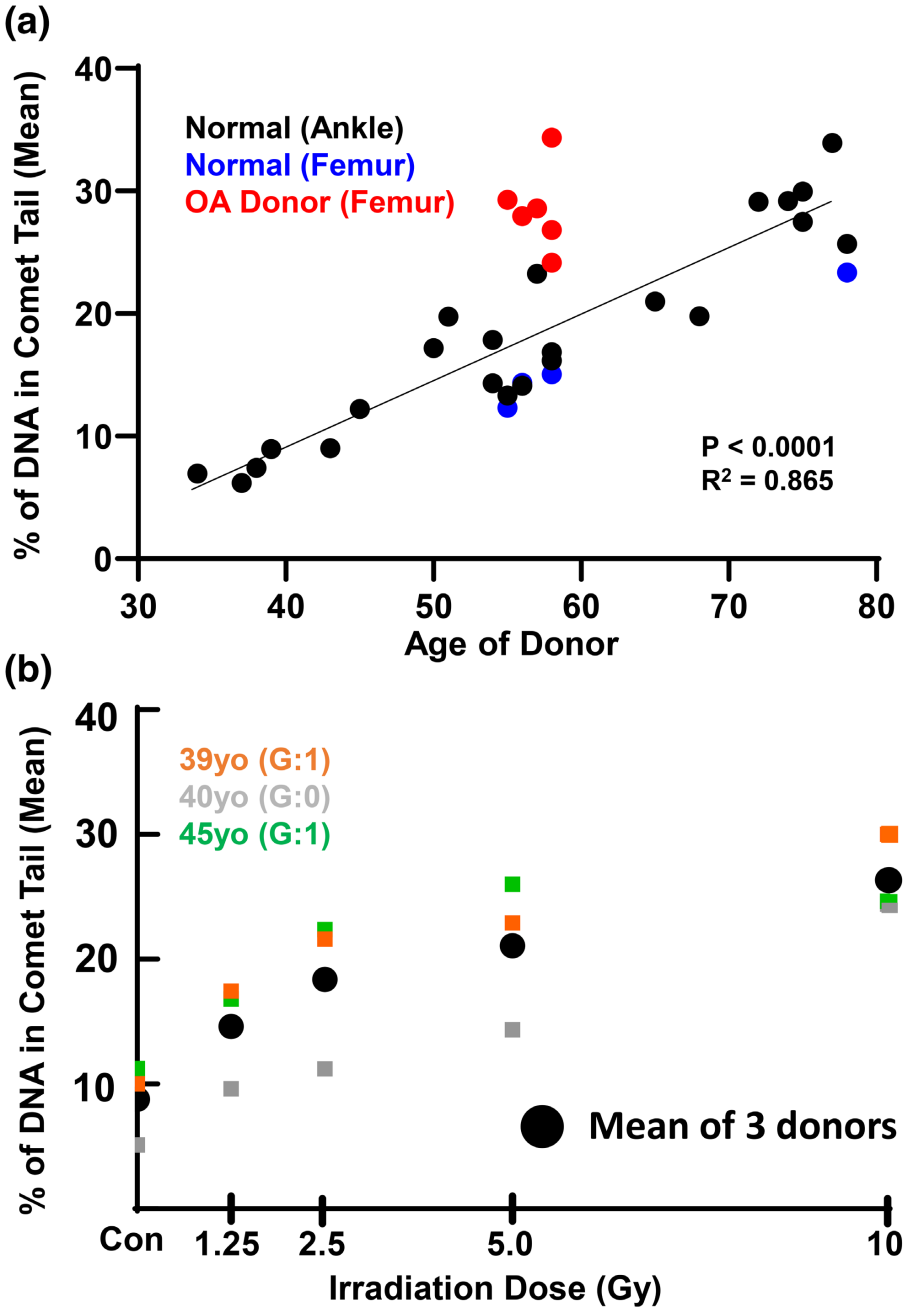
Linearity of DNA damage increase with age and comparison to damage from irradiation. (a) Linear regression for the 23 normal ankles, with p value and R^2^ shown. OA donors (red) are plotted next to the normal donors and fall above the regression line. Cadaveric femur (blue) was available from four donors. Data are from Figure 1b, d, with the addition of 4 donors: 45 yo (grade 1,2), 65 yo (grade 0,1), 68 yo (grade 1,2), and 78 yo (grade 1). (b) DNA damage with increasing irradiation dose from 0 to 10 Gy. The average percent DNA in comet tail from the chondrocytes treated with 10 Gy of irradiation was 26.4%.

Compiling data from the comet assay on cadaveric talar chondrocytes from 25 donors between 34 and 78 years of age revealed a linear increase in the percentage of DNA in the comet tail (Figure 2a, *R* ^2^ = 0.865 by linear regression, *p* < 0.0001). OA chondrocytes plotted on the same figure fall above the trendline (Figure 2a, red dots) and femoral chondrocytes from cadaveric donors fell slightly below the trendline (Figure 2a, blue dots). Of note, the four cadaveric donors in which both ankle and femur cartilage were available demonstrate a similar burden of DNA damage across these two anatomical sites.

### 2.5 High doses of irradiation are required to match the level of age‐associated DNA damage

Chondrocytes of young donors with low baseline levels of damage (39, 40, and 45 years of age) were treated with increasing levels of irradiation: 0 Gy, 1.25 Gy, 2.5 Gy, 5 Gy, and 10 Gy. Cells cultured in well plates were placed directly in a RS2000 Biological Irradiator. Following irradiation, the media was replaced and the cells were allowed to recover to allow for repair of acute DNA damage, with the comet assay performed after 48 h to assess the persistent damage. The irradiated chondrocytes showed a corresponding increase in the level of DNA damage as assessed by the percent of DNA in comet tails (Figure 2b). The chondrocytes from these donors reached an average of 26.4 percent DNA in comet tail at 10 Gy of irradiation. This level of damage is comparable to that found in chondrocytes from either OA donors between the ages of 50–60 years (mean of 28.5% DNA in comet tail) or cadaveric donors between the ages of 70–80 years (mean of 29.9% DNA in comet tail).

## 3 DISCUSSION

This study quantified DNA damage in primary human chondrocytes, with a particular emphasis on the effects of aging and OA. Our goal was to assess baseline damage and minimize the effects of acute changes due to tissue isolation, storage, or enzymatic digestion. Thus, we plated chondrocytes in monolayer to allow for recovery, which also removes dead or dying cells that are unable to successfully plate down. Chondrocytes were then routinely cryopreserved and thawed in batches containing multiple samples from each group (e.g., young and older; normal and OA) to facilitate direct comparisons, with trial experiments confirming that cryopreservation did not significantly alter the level of DNA damage. The single cell electrophoresis comet assay was sufficiently sensitive to detect increased DNA damage in older donors as compared to young. Assessing the percentage DNA in the “comet tail” of donors between the ages of 34 and 78 showed a linear increase with age, although the progression to end‐stage OA appears to accelerate this process. The finding of significant DNA damage in OA chondrocytes is consistent with previous studies in human and porcine cartilage (Chen et al., 2008; Rose et al., 2012). While the increased DNA damage with “normal” aging has not been previously reported for chondrocytes, our results are consistent with studies in peripheral blood mononuclear cells that have shown an increase of ~1% per year in damage by the comet assay (Moller, 2019).

Ionizing irradiation was used to contextualize the extent of damage measured by the comet assay. Remarkably, 10 Gy irradiation was required for young chondrocytes to reach the levels of DNA damage found in aged and OA chondrocytes. This high level of baseline damage is likely to have phenotypic consequences, as we have shown that 10 Gy irradiation is sufficient to induce senescence in human chondrocytes when coupled with growth factor activation (Copp et al., 2021). Markers of senescence increase with age in both human and murine chondrocytes (Diekman et al., 2018), and the presence of senescent cells in the joint has been implicated in OA pathophysiology (Jeon et al., 2017). This is consistent with evidence in other tissue systems that supports DNA damage as a key driver of the cellular dysfunction that emerges during aging (Schumacher et al., 2021; Yousefzadeh et al., 2021). It will be important to further dissect the mechanisms by which chondrocytes accumulate such high levels of damage. For example, the relative contribution of increased susceptibility to damage and slower repair is unknown, but there is evidence in other cell types that aging results in a compromised capacity for repair (Chen et al., 2020). Further, hypo‐replicative cell types such as chondrocytes may downregulate global DNA repair and prioritize the maintenance of genomic regions required for cell identity (Nouspikel & Hanawalt, 2002; Reid et al., 2021; Wu et al., 2021).

## AUTHOR CONTRIBUTIONS

Michaela Copp involved in conceptualization, methodology, formal analysis, investigation, writing–original draft, visualization, and data curation. Susan Chubinskaya involved in conceptualization, resources, writing–review and editing, and project administration. Daniel Bracey involved in conceptualization, resources, and writing–review and editing. Jacqueline Shine involved in methodology and investigation. Garrett Sessions involved in methodology, formal analysis, and data curation. Richard Loeser involved in conceptualization, resources, writing–review and editing, project administration, and funding acquisition. Brian Diekman involved in conceptualization, investigation, writing–original draft, visualization, supervision, project administration, and funding acquisition.

## CONFLICT OF INTEREST

The authors declare that they have no conflict of interest.

## Supporting information


Appendix S1

## Data Availability

The data that support the findings of this study are available from the corresponding author upon reasonable request.
