# Supplementary material for: Comet assay for quantification of the increased DNA damage burden in primary human chondrocytes with aging and osteoarthritis
Source: Aging Cell. 2022 Aug 22;21(9):e13698. doi: 10.1111/acel.13698 (PMC9470893; doi:10.1111/acel.13698)
Supplement: Supplementary file 1 — Appendix S1 [file ACEL-21-e13698-s001.pdf]

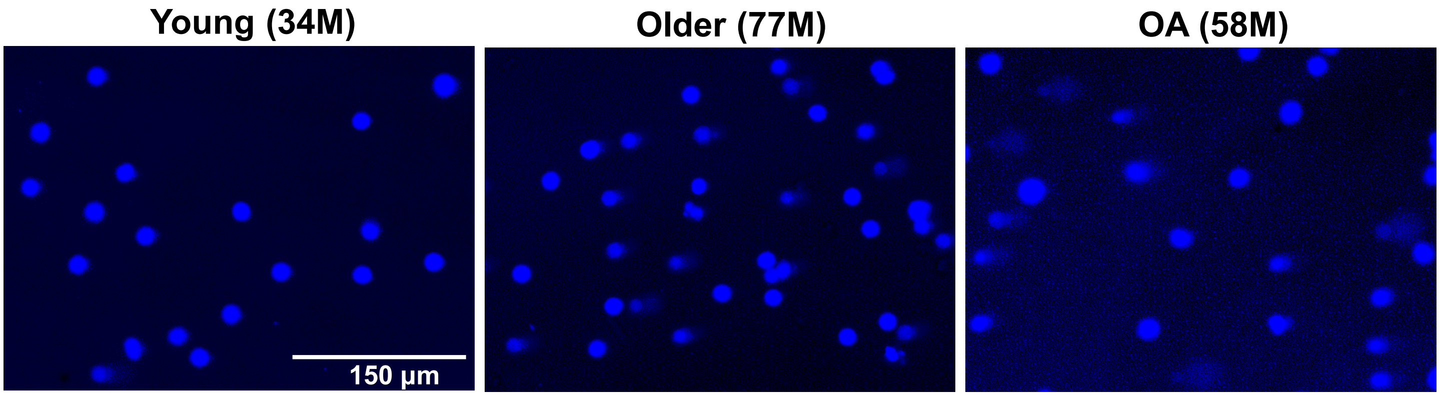

Fig. S1: Representative wide-field images of chondrocyte comets. Chondrocytes from young (34 y.o. male), older (77 y.o. male), and end-stage osteoarthritis (58 y.o. male) were analyzed by alkaline comet assay. Images are representative of those used to quantify the % of DNA in tail in Figure 1.

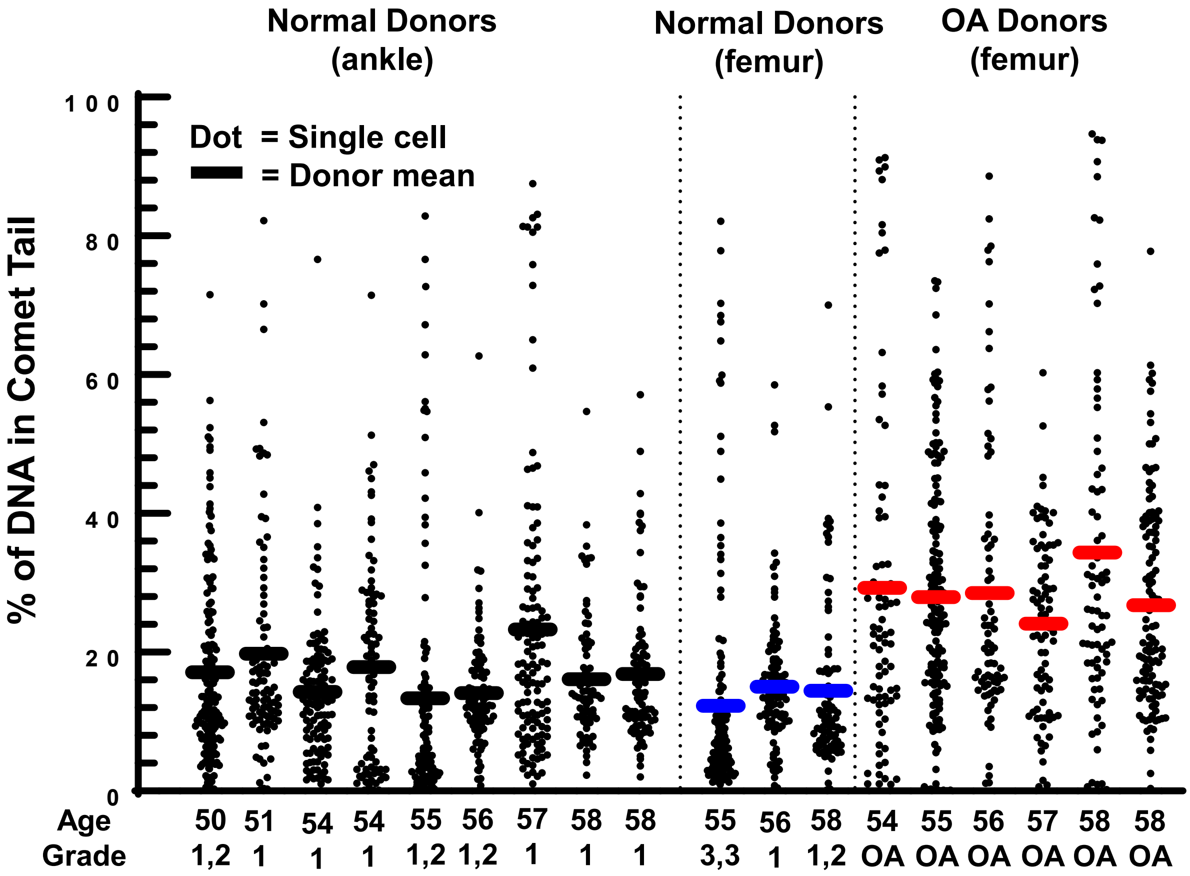

Fig. S2: Individual cell analysis of donors aged 50-60 years. Percent DNA in comet tails from chondrocytes derived from OA femur cartilage compared to cadaveric femur or ankle donors. Age and Collins grade are shown for each donor (multiple numbers indicate a distinct score for the two joints of a given donor, which were combined). The mean % of DNA in comet tail for each donor is plotted in Figure 1D.
